# Mesenchymal Stromal Cells Enhance Vascularization and Epithelialization within 7 Days after Gingival Augmentation with Collagen Matrices in Rabbits

**DOI:** 10.3390/dj9090101

**Published:** 2021-09-04

**Authors:** Anatoliy Kulakov, Evgenia Kogan, Tatiana Brailovskaya, Anna Vedyaeva, Nickolay Zharkov, Olga Krasilnikova, Mikhail Krasheninnikov, Denis Baranovskii, Timur Rasulov, Ilya Klabukov

**Affiliations:** 1 Central Research Institute of Dentistry and Maxillofacial Surgery, 119991 Moscow, Russia; kulakov@cniis.ru (A.K.); brailovskTV@mail.ru (T.B.); vandrer@mail.ru (A.V.); rasulov.doc@gmail.com (T.R.); 2 Strukov Department of Pathological Anatomy, Sechenov First Moscow State Medical University (Sechenov University), 119991 Moscow, Russia; koganevg@gmail.com (E.K.); nickzharkov@mail.ru (N.Z.); 3 Faculty of Dentistry, Sechenov First Moscow State Medical University (Sechenov University), 119435 Moscow, Russia; 4 Department of Regenerative Technologies and Biofabrication, National Medical Research Radiological Center, 249036 Obninsk, Russia; olga4biology@gmail.com (O.K.); doc.baranovsky@gmail.com (D.B.); 5 Research and Educational Resource Center for Cellular Technologies, Peoples’ Friendship University of Russia, 117198 Moscow, Russia; krashen@rambler.ru

**Keywords:** dentistry, gingiva, mesenchymal stromal cells, Mucoderm, Mucograft, stem cell transplantation, tissue engineering

## Abstract

Soft gingival tissue deficiency remains a severe problem leading to postoperative recession, peri-implantitis, and bone resorption. The use of collagen matrices does not always lead to complete rebuilding of the gingiva volume. The application of mesenchymal stromal cells (MSCs) simultaneously with collagen materials represents a promising approach for the restoration of soft gingival tissues. However, short-term effects of MSCs-enriched collagen grafts after gingival augmentation have not yet been studied properly. Mucograft and Mucoderm matrices were implanted in rabbits (n = 12) simultaneously with the intraoperative injection of rabbit bone marrow-derived mesenchymal stromal cells (BM-MSCs) or without cells. Collagen matrices were implanted under the flap or by the surface technique without intentional primary closure. The samples were harvested seven days after implantation, histological staining with hematoxylin and eosin, and immunohistochemical staining for VEGF, IGF1, and TGF were performed. The use of Mucoderm led to better augmentation outcomes on day 7 compared with Mucograft (*p* < 0.0001). Gingival augmentation in combination with the local administration of BM-MSCs led to better regeneration of the soft gingival tissues independently of the type of implanted collagen matrices (*p* < 0.0001). Furthermore, injection of BM-MSCs significantly enhanced gingival vascularization and epithelization with a clear positive correlation between vascular growth and epithelial response. Administration of BM-MSCs in combination with various collagen materials may potentially improve gingiva regeneration.

## 1. Introduction

Dental implantation in patients with a thin gingival phenotype and soft tissue deficiency may lead to postoperative recession and peri-implantitis, accompanied by bone resorption in the neck of the dental implant. In particular, an advanced peri-implant recession occurs in more than 10% of direct implantation cases [1]. Surgical correction is widely used in current clinical practice to increase the alveolar process of soft tissues. However, transplantation of a gingival autograft is quite traumatic for patients and could be associated with additional complications in the donor region. The use of collagen matrices to create the necessary volume of the gingival tissues is a promising approach since it reduces the volume of surgical intervention and traumatization of the donor site [2]. However, most of these membranes effectively perform only barrier functions and do not always significantly increase the soft gingival tissues. Tissue engineering can improve the positive effect of collagen materials allowing efficient recovery of both bone [3] and soft tissue defects [4]. The development of methods and practical recommendations for using autologous mesenchymal stromal cells (MSCs) together with collagen materials to increase the effectiveness of dental implantation is an essential task of dentistry [5]. However, only a few research articles describe the stimulation of gingival soft tissue regeneration using collagen matrices [6].

Mucoderm (Botiss Biomaterials GmbH, Zossen, Germany) and Mucograft (Geistlich Pharma AG, Walhusen, Switzerland) collagen matrices are commonly used to restore the gingival volume [7]. Previous in vitro studies show differences in the mechanical properties, microarchitectonics, and cytocompatibility of Mucoderm and Mucograft materials [8]. At the same time, the difference in the effectiveness of Mucoderm and Mucograft materials for gingival augmentation is poorly understood [9].

The positive impact of cell therapy on gingival regeneration was shown previously. The study by Zanwar et al. (2014) demonstrates that the implantation of synthetic PLA/PGA scaffolds seeded with cells decreases the regeneration time compared with sole autograft [10]. The study by Venkataiah et al. (2019) shows that local administration of allogeneic MSCs from adipose tissue leads to the restoration of the periodontal tissue volume in pigs providing an immunomodulatory effect [11]. Li et al. (2018) showed that implantation of gingival human fetal MSCs helps to restore gingival volume in a rat model [12]. It is clear that regeneration of tissues is based on the activity of a population of tissue and bone marrow stem cells and can be stimulated by fetal MSCs [12]. However, comparative studies show that bone marrow-derived MSCs (BM-MSCs) display more pronounced anti-inflammatory and immunomodulating paracrine activity that can stimulate regeneration [13]. Therefore, BM-MSCs are considered to be the most promising candidates for restoring the structure and function of damaged tissues. 

This work aims to study short-term tissue reactions within gingival restoration by collagen matrices, combined with local injection of BM-MSCs or without cell injection, implanted either by the surface augmentation or under the mucoperiosteal flap.

## 2. Materials and Methods

### 2.1. Laboratory Animals

The experimental study was performed on Gray Giant Flanders rabbits (n = 12) with an initial body weight of 3.0 kg at the age of 0.5–1 year. The animals were kept in the Central Vivarium of the Sechenov University with free access to food and water. All manipulations with laboratory animals were approved by the Local Ethics Committee of the Sechenov University (Protocol Nos. 12–19 of 4 September 2019) and carried out in compliance with the bioethics rules approved by the European Convention for the Protection of Vertebrate Animals used for experiments or other scientific purposes (1986).

### 2.2. Cell Culture 

BM-MSCs were obtained under the standard protocol used in the Shared-Use Facility Center “Regenerative medicine” of Sechenov University for animal BM-MSCs processing [14]. Briefly, rabbit MSCs were isolated from bone marrow, which was obtained by perfusion of the femur. A bone marrow aspirate was placed in a sterile tube containing 50 IU/mL heparin and 0.25 mg/L gentamicin in PBS and delivered to the laboratory at a temperature of +20–24 °C. A 10 mL of bone marrow aspirate was placed in a centrifuge tube and pelleted by centrifugation at 350× *g* for 5 min. The supernatant was removed, the cell pellet was resuspended in 20 mL of lysis buffer (114 mM NH4Cl, 7.5 mM KHCO3, 0.1 mM EDTA) for 3–5 min. After second centrifugation, the cell pellet was resuspended in a nutrient medium for BM-MSCs (DMEM/F12 (Invitrogen, Waltham, MA, USA), 10% FBS (Invitrogen, Waltham, MA, USA), 0.4 μM insulin, 20 ng/mL bFGF, 10 nM dexamethasone, 100 u/mL penicillin, and 100 μg/mL streptomycin (Invitrogen, Waltham, MA, USA)). Cells were stained with a 0.4 Trypan blue solution (Gibco™) and counted using an automatic cell counter Countess™ (Invitrogen™).

The cells were passaged four times according to a common protocol. Briefly: 5–7 mL of a suspension of mononuclear cells (1.0–1.5 × 10^6^ cells/mL) was sown in culture flasks (25 cm^2^) and incubated at 37 °C, 5% CO_2_ under high humidity conditions. The culture medium was changed every 72 h. When 90% confluency was reached, the cells were washed with Dulbecco’s PBS without Ca^2+^ and Mg^2+^ ions (Sigma-Aldrich, Salisbury, UK), then cells were removed from the culture plastic with TrypLe solution (Invitrogen, Waltham, MA, USA), centrifuged at 350× *g* for 5 min, the supernatant was collected, and the pellet was resuspended in a nutrient medium, next 1/3 of the cell suspension was placed on a new culture flask. The cycle was repeated 4 times.

### 2.3. Collagen Matrices

Two types of collagen matrices were used in this study. Mucoderm (Botiss Biomaterials GmbH, Zossen, Germany) is a 3D collagen matrix, a cell-free structure with a surface layer on the upper side of the matrix and a porous internal structure with a parallel arrangement of densely packed bundles consisting of highly purified porcine collagen type I and III without cross-links and elastin. 

Mucograft (Geistlich Pharma AG, Wolhusen, Switzerland) is a 3D collagen matrix consisting of highly purified porcine collagen type I and III without cross-links, containing two layers of dense collagen and a spongy scaffold [15,16,17].

### 2.4. Matrices Implantation and Postoperative Monitoring

According to the type of implanted collagen matrix and implantation method, the rabbits were divided into 4 groups. Each group was divided into 2 subgroups depending on whether the implantation area was injected by BM-MSCs (lower jaw of rabbit) or not (upper jaw of rabbit) (Table 1). 

The surgery was performed under general anesthesia (intramuscular injection of a combination of xylazine hydrochloride (Xyla, Interchemie, Venray, The Netherlands) and tiletamine hydrochloride and zolazepam hydrochloride (Zoletil-100, Virbac, Carros, France)).

**Figure 1:**
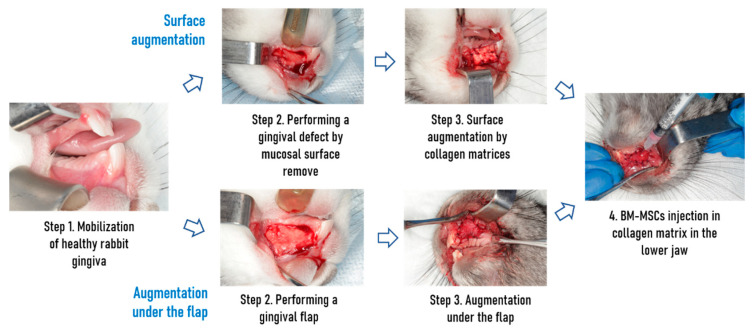
Gingival mucosa reparation pipeline depends on the type of augmentation and presence of cells administration.

Some of the collagen matrices were implanted by the surface augmentation without intentional primary closure on the lateral surface of the jaw. Briefly, the mucous was peeled off and excised, the collagen matrix was fixed to the periosteum. Other collagen matrices were fixed under the mucoperiosteal flap in the area of the incisors of the jaw (Figure 1).

In the subgroups receiving cell injection, BM-MSCs (8 × 10^5^ cells per 0.25 mL of PBS) were injected intraoperatively with an insulin syringe into the area of collagen matrix implantation to ensure cell colonization of both the matrix and the wound surface. Postoperatively, all animals received a subcutaneous injection of enrofloxacin (Baytril, Bayer Animal Health GmbH, Leverkusen, Germany) according to the manufacturer’s protocol for 7 days. Animals were monitored daily for signs of infection or rejection.

### 2.5. Histological Examination

Seven days after implantation, samples were harvested, fixed in 10% neutral buffered formalin, and embedded in paraffin. Serial paraffin sections with a thickness of 4 µm were stained with hematoxylin and eosin (H&E), and van Gieson’s picrofuchsin for further morphological assessment using the scoring method described earlier.

The semi-quantitative scale was used for assessing the regeneration of soft gingival tissue: epithelium (0 points, minor regenerative changes; 1 point, hyperplasia; 2 points, hyperplasia and acanthosis; 3 points, hyperkeratosis); angiogenesis (0 points, absent; 1 point, weak; 2 points, moderate; 3 points, significant); lymphocyte infiltration (0 points, absent; 1 point, weak; 2 points, moderate; 3 points, significant); fibroblastic response (0 points, absent; 1 point, weak; 2 points, moderate; 3 points, significant).

### 2.6. Immunohistochemistry

The presence of IGF1, VEGF, and TGFβ that characterizes the processes of cell proliferation, angiogenesis, and fibrogenesis was determined by immunohistochemistry of harvested samples. On serial paraffin sections, immunohistochemical reactions were performed with mouse monoclonal antibodies to IGF1 (clone W18, dilution titer 1:100, Santa Cruz Biotechnology, Dallas, TX, USA), VEGF (clone VG1, dilution titer 1:100, Agilent/Dako, Santa Clara, CA, USA), rabbit polyclonal antibodies to TGFβ (dilution titer 1:100, Abcam, Cambridge, UK), followed by counterstaining of cell nuclei with Mayer’s hematoxylin. Antigen unmasking was carried out in a citrate-buffered retriever with pH = 6.0 at 98 °C for 20 min, then cooling to 60 °C. Positive and negative control reactions were set. The results of the responses were assessed by a semi-quantitative method described above on a scale from 0 to 3 points.

### 2.7. Microscopy

Following histological and immunohistochemical staining, samples were examined in a light microscopy Carl Zeiss Lab.A1 (Carl Zeiss, Jena, Germany) with digital camera AxioCam ERc5s (Carl Zeiss, Germany) by using the ZEN Lite software (Carl Zeiss, Germany).

### 2.8. Statistics

Statistical analysis of the obtained data was carried out in GraphPad Prism 7.0 software (USA) using a three-way ANOVA with post hoc analysis according to Tukey’s mean. To search for correlations, the methods of nonparametric statistics (Spearman’s rank correlation coefficient) were used. The level of statistical significance was taken as a *p*-value < 0.05.

## 3. Results

### 3.1. Clinical View of the Postoperative Condition of the Gingiva

Gingival Swelling was a common local finding in all groups. The animals had a decreased appetite, which the postoperative pain could explain. 

### 3.2. Morphological Studies

**Figure 2:**
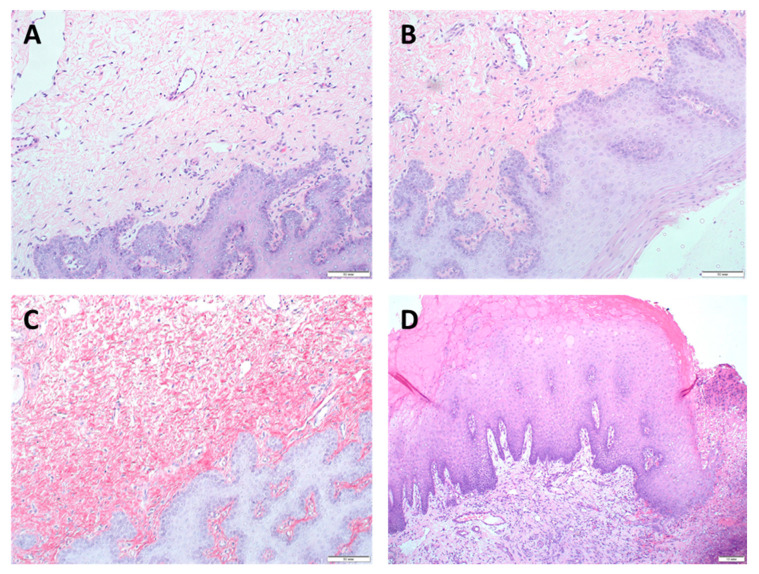
Morphological characteristics of regenerative changes on the 7th day after augmentation of the gingival mucosa with Mucograft material. H&E staining, (**A**–**C**) magn. ×200, scale bar 50 μm; (**D**) magn. ×100, scale bar 10 μm: (**A**) surface augmentation without local injection of MSCs (SGW subgroup)-hyperplasia of the integumentary squamous epithelium with the formation of acanthotic cords, angiogenesis in the form of individual capillary vessels, weak lymphohistiocytic infiltration, and fibroblastic responses were observed; (**B**) surface augmentation in combination with local injection of MSCs (SGI subgroup), the values of all studied parameters tended to increase or were higher compared with SGW subgroup; (**C**) under the flap without local injection of MSCs (UGW subgroup), morphological changes did not differ from those observed in the SGW subgroup implanted with the use of only Mucograft by the surface augmentation; (**D**) under the flap in combination with local injection of MSCs (UGI subgroup), an increase in hyperplasia and acanthosis of the integumentary squamous epithelium, angiogenesis, with a decrease in the intensity of lymphohistiocytic infiltration and fibroblastic responses in comparison with UGW subgroup implanted with the use of only Mucograft without MSCs injection were observed.

All animals had regular food and water intake and survived seven days after implantation. No signs of infection or rejection at the implantation site were observed. Seven days after implantation, animals were sacrificed, and samples were harvested. Histological studies showed that on the 7th day of the experiment complete tissue regeneration occurred in all groups. The morphological characteristics of regenerative changes after augmentation of the gingival mucosa with Mucograft material are shown in Figure 2 and Figure S1 (in Supplementary Materials).

**Figure 3:**
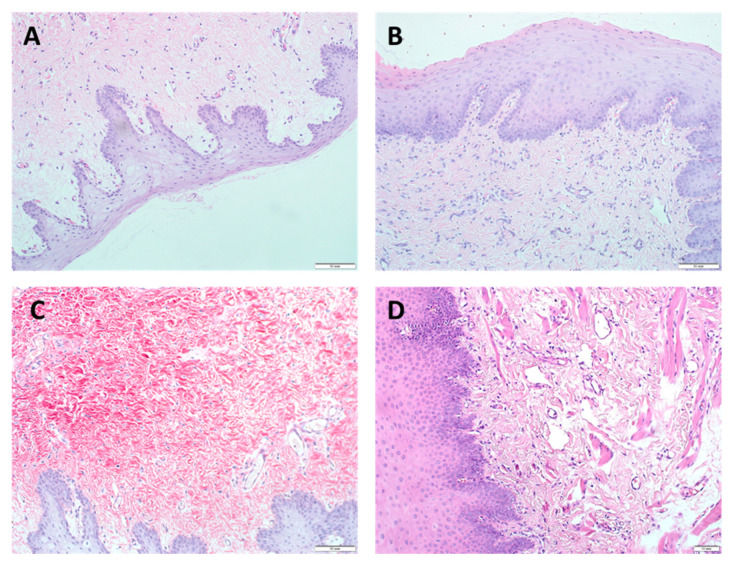
Morphological characteristics of regenerative changes on the 7th day after augmentation of the gingival mucosa with Mucoderm material, H&E staining (**A**–**C**) magn. ×200, scale bar 50 μm; (**D**) magn. ×100, scale bar 10 μm: (**A**) surface augmentation without local injection of MSCs (SDW subgroup), intact integumentary squamous epithelium without signs of regenerative changes, angiogenesis in the form of separate capillary vessels, weak lymphohistiocytic infiltration, and fibroblastic responses were observed; (**B**) surface augmentation in combination with local injection of MSCs (SDI subgroup), signs of regenerative changes in the epithelium, increased angiogenesis, lymphohistiocytic infiltration, and fibrogenesis in the absence of changes in the fibroblastic response were observed; (**C**) under the flap without local injection of MSCs (UDW subgroup), morphological changes did not differ from those observed in the SDW subgroup implanted with the use of only Mucoderm by the surface augmentation; (**D**) under the flap in combination with local injection of MSCs (UDI subgroup), increased hyperplasia with foci of hyperkeratosis and acanthosis of the integumentary squamous epithelium, significantly higher angiogenesis, a decrease in lymphohistiocytic infiltration, and no changes in the fibroblastic responses were observed. A feature of the tissue reaction was the appearance of myofibroblastic foci in the submucosal layer, indicating a high regenerative activity of the tissue, possibly with the participation of MSCs.

The morphological characteristics of regenerative changes after augmentation of the gingival mucosa with Mucoderm material are shown in Figure 3 and Figure S2 (in Supplementary Materials).

### 3.3. Epithelialization Assessment

**Figure 4:**
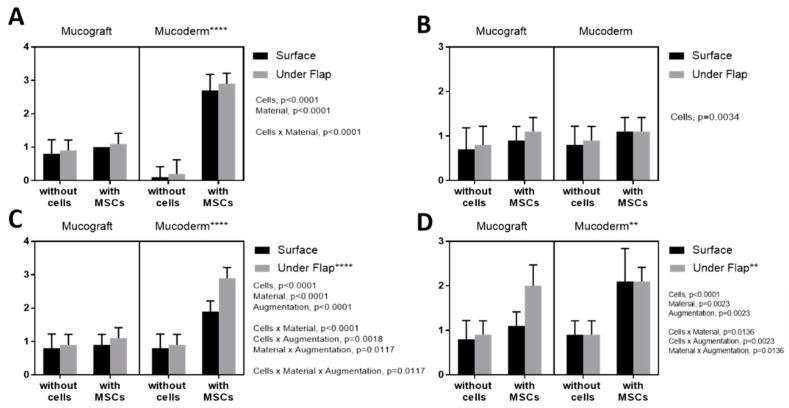
Assessment of epithelialization: (**A**) histological examination, (**B**) stained for IGF; (**C**) stained for VEGF; (**D**) stained for TGFb. **: *p*-value < 0.001, ****: *p*-value < 0.0001.

BM-MSCs injection, as well as the use of the Mucoderm matrix, had a positive effect on epithelialization (Figure 4) (*p*-value < 0.0001). When using Mucoderm with BM-MSCs, the epithelialization values were significantly higher compared with Mucograft (*p*-value < 0.0001), and the method of augmentation had no impact on epithelialization (*p*-value = 0.112). Histology results were confirmed by immunohistochemistry (IHC). However, there was no difference in IGF staining depending on the type of collagen material and implantation methods. There was a positive correlation between the level of angiogenesis and epithelial response (Spearman rank correlation r = 0.786, *p*-value = 0.0006).

### 3.4. Angiogenesis Assessment

**Figure 5:**
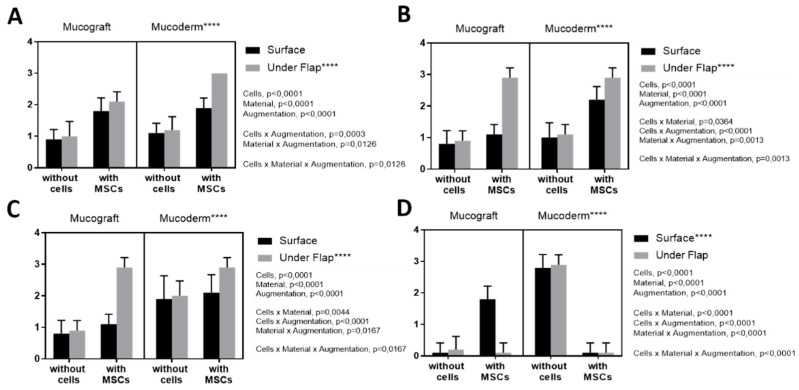
Assessment of angiogenesis (**A**) histological examination, (**B**) stained for IGF; (**C**) stained for VEGF; (**D**) stained for TGFb. ****: *p*-value < 0.0001.

Histological examination showed that the local administration of MSCs had a positive effect on angiogenesis (*p*-value < 0.0001) (Figure 5). Mucoderm had a greater effect compared with Mucograft (*p*-value < 0.0001), as well as the implantation under the flap (*p*-value < 0.0001). The histology results were confirmed by IHC: an increased expression of IGF and VEGF was observed, suggesting an increase in tissue regeneration and angiogenesis processes.

### 3.5. Fibroblast Response Assessment

**Figure 6:**
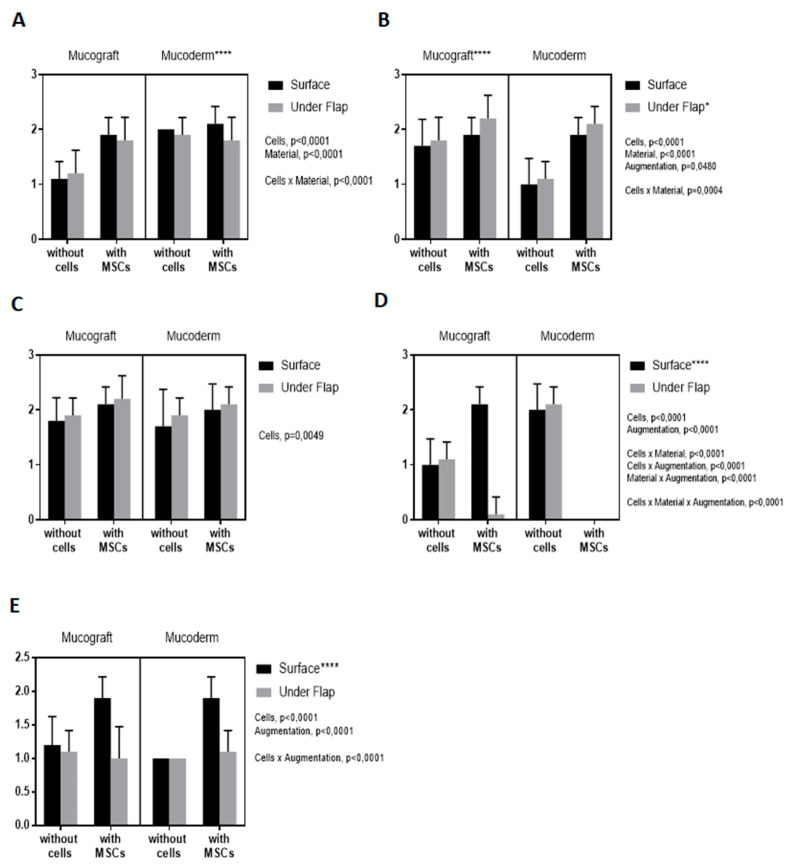
Evaluation of the response of fibroblasts: (**A**) by histological examination, (**B**) when stained for IGF; (**C**) when stained for VEGF; (**D**) when stained for TGFb. Assessment of lymphoid infiltration (**E**) by histological examination. *: *p*-value < 0.05, ****: *p*-value < 0.0001.

Histological examination showed that MSCs injection (*p*-value < 0.0001) and Mucoderm (*p*-value < 0.0001) had a positive effect on the fibroblast response (Figure 6A–D). The method of implantation did not affect the fibroblast response (*p*-value = 0.195).

However, histology results were not confirmed by the IHC. When stained for IGF, Mucograft showed a positive effect. When stained for TGFb, surface augmentation without MSCs administration showed a positive impact on the fibroblast response.

### 3.6. Lymphoid Infiltration Assessment

Histological examination showed that MSCs injection (*p*-value < 0.0001) and the surface augmentation (*p*-value < 0.0001) had a positive effect on lymphoid infiltration (Figure 6E). The type of material had no effect (*p*-value = 0.482). 

## 4. Discussion

The insufficient thickness of the soft gingival tissues is a serious clinical problem that can be solved using various commercially available collagen matrices implanted by different methods. In this work, to identify the most appropriate strategy for increasing the gingival volume, we studied the effectiveness of using two different collagen matrices-Mucoderm and Mucograft, implanted either by the surface augmentation or under the mucoperiosteal flap with the simultaneous local injection of BM-MSCs or without it.

Previous preclinical studies showed that subepithelial connective tissue graft was superior to collagen matrices concerning connective tissue thickness and quality [18]. Consequently, we concluded that the use of collagen matrices requires an additional modification to achieve better results. In the present study, we investigated where local injection of MSCs can enhance the potential of collagen matrices. The research on the use of MSCs has received increased interest due to experimental evidence that MSCs can positively affect the treatment of certain dental diseases, for example, periodontitis [19]. However, the use of cells to stimulate gingival soft tissue augmentation requires the selection of a suitable source of cells, as well as a biologically and physiologically compatible scaffold [12,14]. Previous studies showed that MSCs isolated from the bone marrow contribute to tissue repair in rabbits [20], hence in the present study BM-MSCs were used.

Although there are several scales for assessing gingival regeneration [21,22], these scales are intended for clinical use and do not allow the evaluation of results of experimental studies. Therefore, we proposed an original semi-quantitative scale with an assessment of epithelialization, angiogenesis, lymphoid infiltration, and fibroblastic response by histological and immunohistochemical staining.

Previously, it has been shown that local administration of BM-MSCs is associated with an improvement of angiogenesis and with a decrease of inflammation [23]. In our study, BM-MSCs represented the angiogenic factor, and the groups receiving BM-MSCs had a statistically significantly higher density of blood vessels 7 days post-surgery (*p*-value < 0.0001) compared with groups in which MSCs were not injected. 

Successful gingival regeneration and uncomplicated wound healing require adequate epithelialization of the gingival surface. We observed a positive correlation between the epithelial response and angiogenesis (r = 0.786, *p*-value = 0.0006). Thus, effective epithelialization of the gingiva was associated with a sufficient density of vessels in the subepithelial layer.

The fibroblastic response is most pronounced at the stage of wound remodeling [24]. At the same time, it is known that BM-MSCs have a stimulating effect on fibroblast proliferation. In particular, the conditioned medium from BM-MSCs stimulates the proliferation of gingival fibroblasts in culture [25]. In our study, histological examination showed that the local administration of BM-MSCs had a stimulating effect on the proliferation of fibroblasts. However, when stained for TGFb, a positive influence on the proliferation of fibroblasts was associated with the surface augmentation without the local administration of MSCs.

Decreased infiltration of lymphoid cells is associated with decreased levels of inflammation and reaction of adaptive immunity. Various studies show that the local administration of BM-MSCs has an immunomodulatory effect, reducing infiltration by T-lymphocytes, monocytes, neutrophils, and recruiting regulatory B- and T-lymphocytes [26]. In our study, the local administration of BM-MSCs was associated with increased infiltration by lymphoid cells (*p*-value < 0.0001), which can be explained by the excessive presence of regulatory lymphocytes in the implantation zone. The surface augmentation was also associated with an increase in the inflammatory response (*p*-value < 0.0001), which can be explained by the greater contamination of the wound with the oral microflora.

It is known that BM-MSCs directly exert a therapeutic effect mainly based on their paracrine properties [27]. We found that implantation of collagen matrices in combination with an injection of BM-MSCs as well as the implantation of collagen matrices under the mucoperiosteal flap had a more pronounced effect on regeneration, as evidenced by a higher content of markers of cell regeneration and angiogenesis (IGF1 and VEGF). It can be explained by the fact that when implanted under the flap, there is no intraoperative elimination of cells from the wound. 

Our study is focused on tissue reactions following the gingival restoration with two different commercial materials. We identified the differences histologically and immunohistochemically. An early inflammatory response rises during the first days post-surgery. Thus, the short-term observation period is the reasonable limitation of our study. Early stages of wound healing are the most informative part of tissue repair when inflammation, regeneration, and neo-forming fibrosis can be described contemporaneously as competing processes. Observed short-time effects of MSC’s cells on inflammation and angiogenesis could potentially be important for elderly patients and patients with diabetes.

Also, our study does not compare the effects of MSCs from other sources. Bone marrow-derived MSCs are well-known and widely accepted due to their effectiveness in regenerating both bone and soft tissues. This effect is based on the cell’s paracrine activity and leads to decreased inflammation and increased vascularization. Clinical administration of BM-MSCs is used for skin burns [28], airway inflammation [29], and diabetic foot ulcers [30]. It has previously been shown that gingival material sampling is often infected in contrast to bone marrow-derived cells, so BM-MSCs may be immediately administered without long-term processing. Targeting a minimal use of surgical activities on the recipient’s gingiva before implantation, we decided to use BM-MSC instead of gingival cells for this study. Gingival tissue sampling for further MSCs isolation will lead to gingival tissue loss in the donor area and provide no clear benefit compared with well-known gingival autograft implantation.

At the same time, the collagen matrix Mucoderm had a more expressed effect on gingival tissue regeneration than Mucograft, evidenced by histological examination showing an increase in cell proliferation and angiogenesis and further confirmed by immunohistochemical staining, that revealed abounded expression of IGF1 and VEGF. At the same time, the differences between the physical properties of Mucoderm and Mucograft collagen matrices were previously noted [15,16]. However, differences in the biochemical composition and cytotoxic properties of these materials were not previously identified. We assume that the pronounced reaction to Mucoderm may be explained by the peculiarities of processing xenogenic material, particularly by the effect of detergents used for decellularization of the biomaterial and by the technological features of lyophilization and preservation during manufacturing.

We also demonstrated that local injection of BM-MSCs contributes to better gingival regeneration, which most likely is the result of the activation of host tissue stem cells. The statistically significant differences between groups were not affected by the fact that each animal was implanted with two samples of a matrix. Matrices were implanted on different jaws of the animal, so the injected BM-MSCs produced only a local effect. 

Nevertheless, correlations between different combinations of collagen matrices and cell injections with late complications remain unclear. Further chronic experiments with a significant number of animals and extended postoperative observation periods could be conducted to clarify the remaining gaps.

## 5. Conclusions

Mucoderm collagen matrix promotes better augmentation outcomes on the 7th day of observation compared with Mucograft material. Augmentation of the soft tissues of the gingiva in combination with BM-MSCs injection promotes better regeneration of the soft gingival tissues. Moreover, the implantation of collagen matrices under the mucoperiosteal flap leads to better augmentation outcomes. At the same time, the combination of the type of collagen material, method of implantation, and injection of BM-MSCs that will show the highest effectiveness in the long term needs to be found in further studies.

## Figures and Tables

**Table 1:** Groups of experimental animals.

Group	Subgroups	
	Without Cells (W)	Injection of MSCs (I)
SG (n = 3)	SGW Subgroup: Surface augmentation (S) Mucograft (G)	SGI Subgroup: Surface augmentation (S) Mucograft (G)
SD (n = 3)	SDW Subgroup: Surface augmentation (S) Mucoderm (D)	SDI Subgroup: Surface augmentation (S) Mucoderm (D)
UG (n = 3)	UGW Subgroup: Augmentation under the flap (U) Mucograft (G)	UGI Subgroup: Augmentation under the flap (U) Mucograft (G)
UD (n = 3)	UDW Subgroup: Augmentation under the flap (U) Mucoderm (D)	UDI Subgroup: Augmentation under the flap (U) Mucoderm (D)

## Data Availability

Data is contained within the article or supplementary material.

## Supplementary Materials

The following are available online at https://www.mdpi.com/article/10.3390/dj9090101/s1, Figure S1: Immunohistochemical characterization of rabbit’s gingiva augmented with Mucograft and stained for IGF1, VEGF, and TGFβ, magn. ×200; Figure S2: Immunohistochemical characterization of rabbit’s gingiva augmented with Mucoderm and stained for IGF1, VEGF, and TGFβ, magn. ×200.
