# Supplementary material for: Mesenchymal Stromal Cells Enhance Vascularization and Epithelialization within 7 Days after Gingival Augmentation with Collagen Matrices in Rabbits
Source: Dent J (Basel). 2021 Sep 4;9(9):101. doi: 10.3390/dj9090101 (PMC8469508; doi:10.3390/dj9090101)
Supplement: Supplementary file 1 [file dentistry-09-00101-s001.zip › dentistry-1315102-supplementary.pdf]

dentistry journal

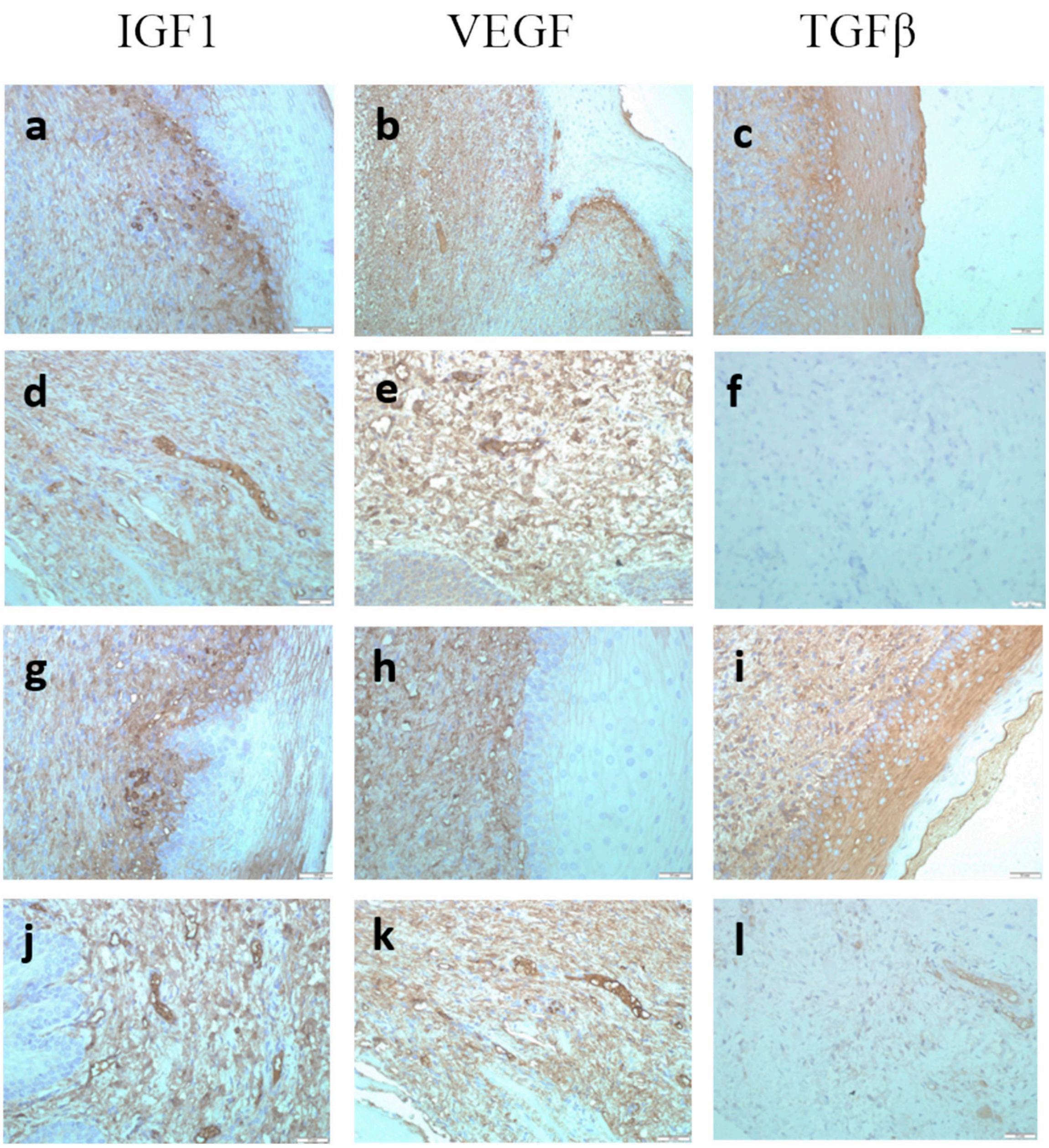

Figure S1. Immunohistochemical characterization of rabbit's gingiva augmented with Mucograft and stained for IGF1, VEGF, and TGFβ, magn. × 200. (**a**)—(**c**) surface augmentation without local injection of MSCs (SGW subgroup): low expression of IGF1, VEGF, and TGFβ was found in the integumentary squamous epithelium, vessels, and fibroblastic elements of the submucosa; (**d**)—(**f**) surface augmentation in combination with local injection of MSCs (SGI subgroup): the values of all studied markers tended to increase, the levels of IGF1, VEGF, TGFβ were higher compared to the SGW subgroup; (**g**)—(**i**) augmentation under the flap without local injection of MSCs (UGW subgroup): the levels of IGF1, VEGF, TGFβ did not differ from those observed in the SGW subgroup; (**j**)—(**l**) augmentation under the flap with local injection of MSCs (UGI subgroup): increased expression of IGF1 and VEGF in the cells of the vascular wall, with a constant level of expression in the integumentary epithelium in comparison with UGW subgroup. On the contrary, the content of TGFβ increases in the epithelium, which may be associated with the phenomena of hyperkeratosis, with a simultaneous decrease in the vessels and fibroblastic elements.

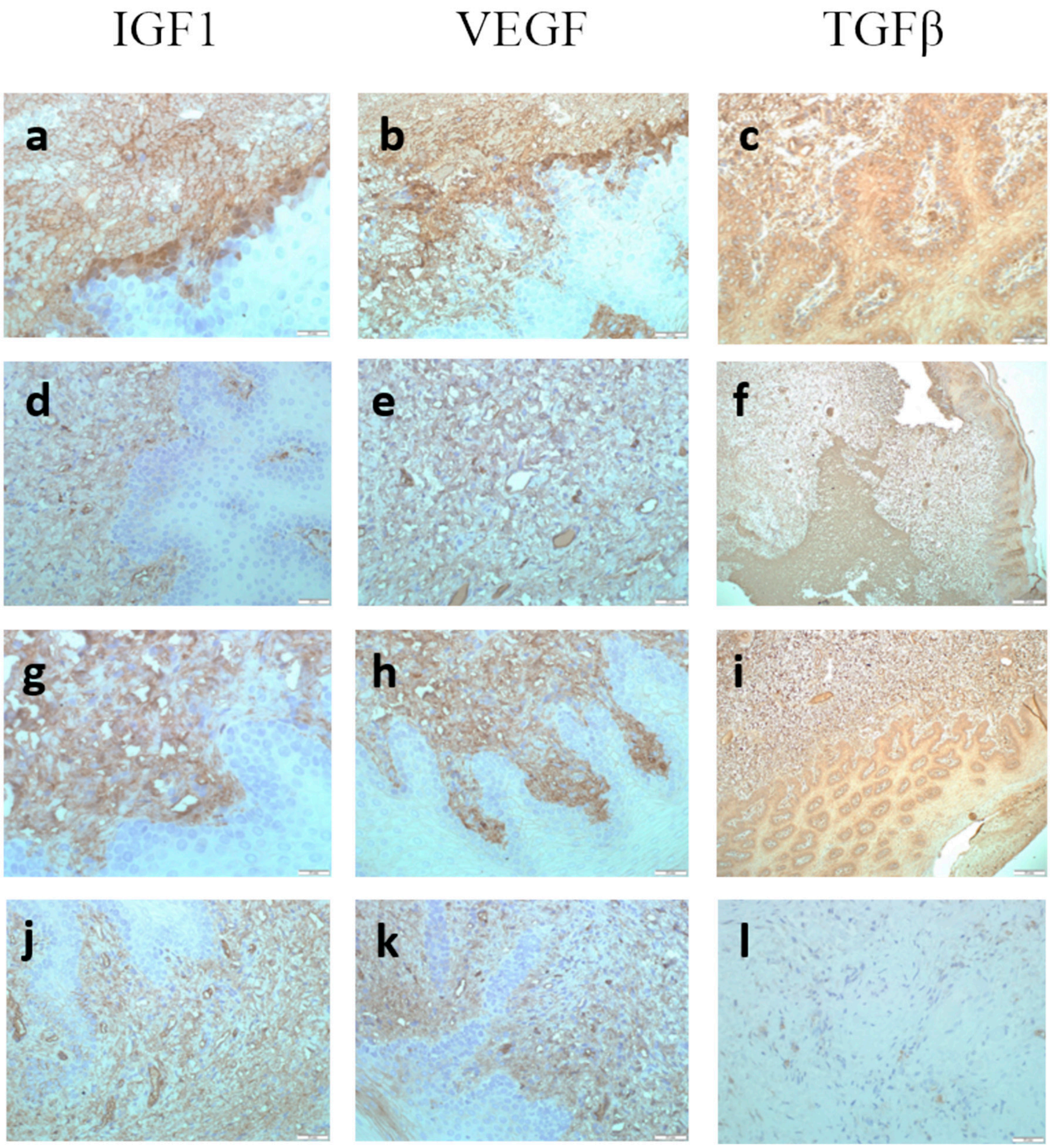

Figure S2. Immunohistochemical characterization of rabbit's gingiva augmented with Mucoderm and stained for IGF1, VEGF, and TGFβ, magn. × 200. (**a**)—(**c**) surface augmentation without the local injection of MSCs (SDW subgroup): a low expression of IGF1 in the epithelium, moderate in the vessels and fibroblastic elements of the submucosal layer, VEGF and TGFβ - low expression in the epithelium and moderate in the cells of the vascular wall and fibroblastic elements; (**d**)—(**f**) surface augmentation in combination with local injection of MSCs (SDI subgroup): the values of all studied parameters tended to increase: IGF1 content was higher in vascular cells and fibroblastic elements, VEGF and TGFβ were higher only in epithelial cells compared to the SDW subgroup. TGFβ was found in small amounts in the cells of the vascular wall and fibroblastic elements; (**g**)—(**i**) augmentation under the flap without the local injection of cells (UDW subgroup): IGF1, VEGF, TGFβ levels did not differ from those observed in the SDW subgroup; (**j**)—(**l**) augmentation under the flap with MSCs injection (UDI subgroup): increased expression of IGF1 and VEGF in the cells of the vascular wall and fibroblastic elements, with a constant level of expression in the integumentary epithelium. In addition, the expression of VEGF and TGFβ is increased in the epithelium, which may be associated with increased angiogenesis and hyperkeratosis.
